# Editorial: Deep neural network based decision-making interpretability

**DOI:** 10.3389/fncom.2023.1276998

**Published:** 2023-09-01

**Authors:** Guitao Cao, Ye Duan, Wenming Cao

**Affiliations:** ^1^Software Engineering Institute, East China Normal University, Shanghai, China; ^2^School of Computing, Clemson University, Clemson, SC, United States; ^3^College of Information Engineering, Shenzhen University, Shenzhen, China

**Keywords:** deep learning, decision model, interpretability, decision-making process, *ante-hoc* interpretability, *post-hoc* interpretability

Deep Neural Networks (DNNs) have emerged as a powerful tool, capable of making complex decisions that were once the exclusive domain of human cognition. However, as these systems become increasingly integrated into our daily lives, the question of their interpretability—the ability to understand and explain their decision-making processes has become a pressing concern. This editorial delves into this Research Topic, and based on existing findings, analyzes and summarizes the contributions of articles within this topic to the research objective.

Model interpretability refers to the model's capacity to elucidate or present complex concepts in a human-readable manner, enabling comprehension by individuals. The interpretability of machine learning models can be broadly categorized into *ante-hoc* interpretability and *post-hoc* interpretability.

*Ante-hoc* interpretability refers to the ability of a model to be interpretable in itself by training simple structures or self-explanatory models that are inherently interpretable. The inherent interpretability of neural network models can be achieved by introducing attention mechanisms. Attention mechanisms have good interpretability since the attention weight matrix directly reflects the areas of interest to the model during the decision-making process. With attention mechanism, Geng et al. propose a novel network, SFLCA-Net, for tic recognition. SFLCA-Net uses two fast and slow branch subnetworks and a light-efficient channel attention (LCA) module. It focuses on capturing the characteristics of fine tic movements and improving the complementarity of spatial-temporal channel information, introducing an attention module to enhance decision-making.

Existing research also achieve model's inherent interpretability by directly incorporating interpretability into specific model structures. Lilan and Yongsheng utilize multi-label classification and a dual-component decision-making process to build a decision-making model, presenting a way to interpret the categorization. The authors theoretically verify the efficiency of their control strategy. This helps to establish a transparent understanding of the model's decision-making process. He, Zhang et al. propose a novel two-stage training method consisting of an initial prediction stage and a fine-tuning stage. The authors introduce CT-GCL, which are used in the fine-tuning stage to reconstruct the sequence in a causal, temporal order. The causal nature of these layers can help to explain the sequence of decisions made by the model, contributing to its interpretability. Zhang and Liu focuses on few-shot learning, providing a detailed taxonomy of few-shot classification methods, comparing these methods, and discussing the scenarios. The authors propose a detailed taxonomy of few-shot classification methods, classifying them into four categories: data augmentation, metric-based methods, optimization methods, and model-based methods. This taxonomy provides a structured way to understand the different strategies employed for few-shot learning, supporting the interpretability research of these models. Li et al. introduce a novel Long-and Short-term Time-series network that utilizes geometric algebra (GA-LSTNet) for the analysis of multi-dimensional time-series (MTS) data. This novel approach, which treats multi-dimensional data as GA multi-vectors, aims to preserve the correlation among different dimensions. The use of geometric algebra adds a layer of interpretability to the model's decisions by offering a mathematical framework that captures the inherent structures and relationships in the data. He, Zheng et al. present an innovative Hough matching feature enhancement network designed to address object counting in a few-shot scenario. This approach uses a Hough space to vote for candidate object regions, resulting in reliable similarity maps between exemplars and the query image. It enhances the interpretability of the model's decision-making process by providing a clear, geometric-based mechanism for how candidate object regions are identified and selected. Usman and Zhong provide a comprehensive survey of the field of human motion prediction, particularly focusing on the use of 3D skeleton data. This review provides insight into the different approaches and techniques of various deep neural networks, and how they contribute to the decision-making interpretability.

*Post-hoc* interpretability occurs after the model training. For a given well-trained deep learning model, *post-hoc* interpretability aims to utilize explanatory methods or construct interpretive models to explain the functioning, decision behavior, and decision rationales of the learned model. Aamir et al. propose a layer-wise analysis approach to determine influence scores and identify influential training images that contribute to class prediction. This technique offers a granular view of how different layers of the convolutional neural network (CNN) model influence the final decision. By analyzing the influence of both training and testing data on the model's decisions, this approach provides a more comprehensive understanding.

## Author contributions

GC: Writing—original draft, Writing—review and editing. YD: Writing—review and editing. WC: Writing—review and editing.

